# Molecular Characterization of a Highly-Active Thermophilic β-Glucosidase from *Neosartorya fischeri* P1 and Its Application in the Hydrolysis of Soybean Isoflavone Glycosides

**DOI:** 10.1371/journal.pone.0106785

**Published:** 2014-09-04

**Authors:** Xinzhuo Yang, Rui Ma, Pengjun Shi, Huoqing Huang, Yingguo Bai, Yaru Wang, Peilong Yang, Yunliu Fan, Bin Yao

**Affiliations:** 1 Key Laboratory for Feed Biotechnology of the Ministry of Agriculture, Feed Research Institute, Chinese Academy of Agricultural Sciences, Beijing, China; 2 Biotechnology Research Institute, Chinese Academy of Agricultural Sciences, Beijing, China; University of Melbourne, Australia

## Abstract

Isoflavone occurs abundantly in leguminous seeds in the form of glycoside and aglycone. However, isoflavone glycoside has anti-nutritional effect and only the free type is beneficial to human health. In the present study we identified a β-glucosidase from thermophilic *Neosartorya fischeri* P1, termed NfBGL1, capable of efficiently converting isoflavone glycosides into free isoflavones. The gene, belonging to glycoside hydrolase family 3, was successfully overexpressed in *Pichia pastoris* at high cell density in a 3.7-l fermentor. Purified recombinant NfBGL1 had higher specific activity (2189±1.7 U/mg) and temperature optimum (80°C) than other fungal counterparts when using *p*-nitrophenyl β-d-glucopyranoside as the substrate. It retained stable at temperatures up to 70°C and over a broad pH range of 3.0−10.0. NfBGL1 had broad substrate specificity including glucosidase, cellobiase, xylanase and glucanase activities, and displayed preference for hydrolysis of β-1,2 glycosidic bond rather than β-1,3, β-1,4, β-1,6 bonds. The enzyme showed high bioconversion ability for major soybean isoflavone glycosides (daidin, gensitin and glycitin) into free forms. These properties make NfBGL1 potential for the wide use in the food, feed, pharmacy and biofuel industries.

## Introduction

Plant isoflavones are important secondary metabolites that are mainly distributed in leguminous plants with high levels [1]. It can occur in glycoside and free types. Most of isoflavones exist in natural materials as glycoside, which represent one of the anti-nutritional factors that reduce the nutrient utilization and/or food intake of plants or plant products as human foods or animal feeds [2], while their free form plays the major beneficial roles to prevent certain cancers, osteoporosis and cardiovascular disease [3]–[5]. Therefore, how to convert isoflavone glycosides to free isoflavones is the key for extensive utilization of isoflavone in the food and feed industries.

β-Glucosidases (EC3.2.1.21) catalyzes the hydrolysis of β-glucosidic linkages of various oligosaccharides and glycosides to release glucose and debranched oligosaccharides. According to the CAZy database (http://www.cazy.org) [6], these enzymes are grouped into 6 of 133 glycoside hydrolase (GH) families, i.e. 1, 3, 5, 9, 30 and 116. β-Glucosidase plays a vital role in the biological conversion of cellulose by degrading the cellooligosaccharide products released by endoglucanase and cellobiohydrolase to form glucose and a debranched oligosaccharide. To avoid product inhibition, β-glucosidase with high tolerance for glucose is heated in these fields [7]. Recently, β-glucosidase has attracted much interest due to its important roles in the hydrolysis of isofalvone glycosides. Those from *Dalbergia* sp. [8], *Paecilomyces thermophila* [9], *Dictyoglomus turgidum* [10], *Sulfolobus solfataricus* [11], and *Rhizopus* spp. [12] have shown ability to degrade isofalvone glycosides. However, these β-glucosidases have low enzymatic activities and low commercial values. What’s more, their conversion efficiencies of glucosidic isoflavones were further reduced due to their sensitivity to glucose inhibition. Thus a highly efficient and glucose-tolerate β-glucosidase with isofalvone glycoside hydrolysis ability is mostly urgently required.

Thermophilic/thermostable enzymes are favorable for hydrolysis of isoflavone glycosides since high temperatures enhance the mass transfer rate, reduce the substrate viscosity, and reduce the risk of contamination [13]. The present study aims to obtain a new thermophilic β-glucosidase for efficient hydrolysis of isoflavone glycosides and other saccharides. A family 3 β-glucosidase, termed NfBGL1, was identified in the thermophilic fungal strain *Neosartorya fischeri* P1 and overexpressed in *Pichia pastoris*. This enzyme was characterized to be thermophilic and highly active and have wide substrate specificity and high soybean isoflavone glycoside-hydrolysis ability. Its supplementation significantly enhanced the conversion of isoflavone glycosides to aglycone types.

## Methods

### 2.1. Materials

Soybean flour extract was prepared following the method of previous report with some modifications [14]. The soybean flour was defatted in three volumes of *n*-hexane for 30 min with agitation at room temperature, centrifuged at 13,000 *g* for 20 min, and air-dried to a fine powder.

The substrates cellobiose, carboxymethyl cellulose sodium (CMC-Na), Avicel, laminiarin, lichenin, amygdalin, daidzin, genistin, glycitin, daidzein, genistein, glycitein, birchwood xylan, beechwood xylan, sophorose, gentiobiose, salicin, lactose, *p*-nitrophenyl β-d-glucopyranoside (*p*NPG) and other *p*-nitrophenyl glycosides, and Novozyme 188 (cellobiase from *Aspergillus niger*) were purchased from Sigma-Aldrich (St. Louis, MO). Other substrates including barley β-glucan and xyloglucan were obtained from Megazyme (Wicklow, Ireland). T4 DNA ligase and restriction endonucleases were purchased from Promega (Madison, WI).

The Fungal DNA Isolation Kit and RNeasy Plant Mini Kit were obtained from Omega Bio-tek (Norcross, GA) and QIAGEN (Hilden, Germany), respectively. The DNA Purification Kit, LA Taq DNA polymerase and restriction endonucleases were purchased from TaKaRa (Otsu, Japan). All other chemicals were of analytical grade and commercially available.

### 2.2. Strains

*N. fischeri* P1 CGMCC 3.15369 [15] was grown in a β-glucosidase inducing medium containing 10 g/l wheat bran, 10 g/l CMC-Na, 5 g/l (NH_4_)_2_SO_4_, 1 g/l KH_2_PO_4_, 0.5 g/l MgSO_4_·7H_2_O, 0.2 g/l CaCl_2_, and 10 mg/l FeSO_4_·7H_2_O at 45°C and pH 5.0 for 4 days. The total β-glucosidase activity in *N. fischeri* culture supernatants was 11.9±0.2 U/ml.

*Escherichia coli* Trans1-T1 (TransGen, Beijing, China) was cultivated in Luria-Bertani (LB) medium with 100 µg/ml ampicillin at 37°C for gene cloning and sequencing. *P. pastoris* GS115 (Invitrogen, Carlsbad, CA) cultivated in yeast peptone dextrose (YPD) medium at 30°C was used for heterologous protein expression. The plasmids pEASY-T3 (TransGen) and pPIC9 (Invitrogen) were used as cloning and expression vectors, respectively.

### 2.3. Cloning and sequence analysis of the β-glucosidase gene (*Nfbgl1*)

Mycelia of strain P1 were collected after 4 days’ growth in β-glucosidase producing medium as described above. Total DNA and RNA were extracted and purified according to the manufacturer’s instructions, respectively. The cDNAs were synthesized *in* *vitro* using the ReverTra Ace-α- Kit (TOYOBO, Osaka, Japan) with the total RNA as the template. According to the genome sequence of a hypothetical β-glucosidase from *N. fischeri* NRRL 181 (XP_001261562), two specific primer sets (Nfbgl1-F: ATGCAGAACCTCTTCCTTTCTCTCC and Nfbgl1-R: CTTTCAGGGTCCGTCGCGGATGGTGA; and Nfbgl1-expF: GGGGAATTCTACGGTTCTGGTGGCAGCAACTGGG and Nfbgl1-expR: GGGGCGGCCGCTCACCATCCGCGACGGACCCTGAAAG, restriction sites underlined) were designed to amplify the full-length β-glucosidase gene, *Nfbgl1*, and its cDNA without the signal peptide coding sequence. The PCR products with appropriate size were ligated into a pGEM-T3 Easy vector (TransGen) for sequencing.

The nucleotide sequence was analyzed using Vector NTI Advance 10.0 software (Invitrogen). BLASTn and BLASTp programs (http://www.ncbi.nlm.nih.gov/BLAST/) and AlignX from Vector NTI were used to analyze the nucleotide and deduced amino acid sequences, respectively. The online software FGENESH (http://linux1.softberry.com/berry.phtml) was used to predict the transcription initiation sites, introns and exons. NetNGlyc 1.0 Server (http://www.cbs.dtu.dk/services/NetNGlyc/) was used to predict the potential *N*-glycosylation sites. Multiple sequence alignment of deduced NfBGL1 and other fungal counterparts were conducted using the ClustalW program (www.genome.jp/tools/clustalw/). The three-dimensional structure was predicted using the SWISS-MODEL (http://swissmodel.expasy.org/) with the GH3 β-glucosidase AaBGL1 from *Aspergillus aculeatus* (PDB: 4IIB; 44.1% identity) [16] as the template.

### 2.4. Heterologous expression and purification of NfBGL1

Heterologous expression of recombinant NfBGL1 was conducted following the *Pichia* Expression Kit (Invitrogen). The cDNA fragment of mature NfBGL1 without the signal peptide-coding sequence was digested with *EcoR*I and *Not*I, and then cloned into the corresponding sites of vector pPIC9. The recombinant plasmid pPIC9-*Nfbgl1* was then linearized with *Bgl*II and transformed into *P. pastoris* GS115 competent cells by electroporation (Bio-Rad, Hercules, CA). The positive transformants were selected based on their enzymatic activities in shake tubes. The transformant that exhibited the highest β-glucosidase activity in culture supernatant was used for large-scale fermentation in 1-l Erlenmayer flasks. The cell-free culture supernatants were collected and loaded onto a HiTrap Q Sepharose XL FPLC column (GE Healthcare, Uppsala, Sweden) equilibrated with buffer A (20 mM McIlvaine buffer, pH 7.0). Proteins were eluted with NaCl gradients (0–1.0 M) in the same buffer at a flow rate of 3 ml/min. The fractions containing enzyme activity were collected and evaluated by sodium dodecyl sulfate-polyacrylamide gel electrophoresis (SDS-PAGE). The protein concentration was determined by the Bradford assay with bovine serine albumin as the standard.

### 2.5. High-cell-density fermentation in a 3.7-l fermentor

The positive transformant with the highest β-glucosidase activity in shake flask culture was selected for high-cell-density fermentation in a 3.7-l stirred-tank fermentor (Bioengineering KLF 2000, Wald, Switzerland) using a three-step fermentation strategy as described [17]. The inoculum ratio was 10% (v/v). The temperature and initial pH was set to be 30°C and 5.0. During the whole fermentation process, samples were periodically withdrawn for analysis of wet weights and enzyme activities.

### 2.6. Enzyme activity assays

The β-glucosidase activity was assayed using *p*NPG as the substrate. The reaction system consisted of 400 µl of 1.25 mM *p*NPG in McIlvaine buffer (200 mM Na_2_HPO_4_, 100 mM citric acid, pH 5.0) and 100 µl of appropriately diluted enzyme solution. The mixture was incubated at 80°C for 10 min, and then terminated by addition of 1.5 ml of 1 M Na_2_CO_3_. After cooling down to room temperature, the absorbance was measured at 405 nm. One unit of enzyme activity was defined as the amount of enzyme that released 1 µmol of *p*-nitrophenol per min. Each reaction was performed in triplicate, and the data were shown as mean ± S.D. (*n* = 3). The enzymatic activities towards other *p*-nitrophenyl glycosides were assayed as described above with different substrates.

Cellulase/glucanase/xylanase activity was determined according to the 3,5-dinitrosalicylic acid (DNS) assay [18]. The standard reaction system containing 100 µl of appropriately diluted enzyme and 900 µl of 1% (w/v) substrate (CMC-Na, Avicel, xyloglucan, barley β-glucan, beechwood xylan or birchwood xylan) in McIlvaine buffer (pH 5.0) was incubated at 80°C for 10 min, followed by addition of 1.5 ml of DNS reagent. After 5-min boiling and cooling down to room temperature, the absorbance was measured at 540 nm. One unit of cellulase/glucanase/xylanase activity was defined as the amount of enzyme that released 1 µmol of reducing sugar per min under the assay conditions.

The enzymatic activities on 10 mM of cellobiose, salicin, lactose, daidzin, glycitin, and genistin were estimated by measuring the amount of released glucose using GOD-POD method with a commercial kit (Biosino, Beijing, China). The absorbance was measured at 505 nm. One unit of enzyme activity was defined as the amount of enzyme that released 1 µmol of glucose per minute.

### 2.7. Biochemical characterization

The optimal pH values were determined at 80°C in the following solutions, 100 mM glycine-HCl for pH 2.0–3.0, McIlvaine buffer for pH 3.0–8.0, 100 mM Tris-HCl for pH 8.0–9.0, and 100 mM glycine-NaOH for pH 9.0–12.0. The pH stability was determined by measuring the residual enzyme activity under the standard conditions (pH 5.0, 80°C, 10 min) after pre-incubation of the enzyme in buffers of pH 2.0–12.0 at 37°C for 1 h without the substrate.

The optimal temperature was examined at optimal pH by measuring the enzyme activity over the temperature range of 20 to 90°C. The thermostability assay was carried out by incubating the enzyme at optimal pH and at 70°C or 80°C without substrate for 2, 5, 10, 20, 30, or 60 min, and then measuring the residual enzyme activities under the assay conditions.

To investigate the effects of different metal ions and chemical reagents on the activity of purified recombinant NfBGL1, the reaction system containing 5 mM of each Na^+^, K^+^, Li^+^, Ag^+^, Cu^2+^, Ni^2+^, Mn^2+^, Ca^2+^, Pb^2+^, Co^2+^, Zn^2+^, Mg^2+^, Fe^3+^, Cr^3+^, SDS, EDTA, and β-mercaptoethanol was subject to enzyme activity assay under the standard conditions and compared to the blank control without any additives.

### 2.8. Substrate specificity and kinetic parameters

The substrate specificities of NfBGL1 were determined under standard assay conditions (pH 5.0 and 80°C for 10 min) in the presence of 1 mM of *p*-nitrophenyl derivatives and oligosaccharides, or 10 mg/ml of polysaccharides and isoflavone glycosides.

The *K*_m_ and *V*_max_ values of NfBGL1 were determined in McIlvaine buffer (pH 5.0) containing 0.01–2 mM *p*NPG at 80°C for 5 min. The experiments were carried out three times, and each experiment included three replicates. The data were calculated using the Lineweaver-Burk method with a non-linear regression computer program GraFit (Version 7.0, Erithacus Software, Horley, UK).

### 2.9. Tolerance to glucose inhibition

The inhibitory effect of glucose on NfBGL1 activity was determined by fitting to the Dixon plot [19], [20]. Aliquots of enzyme solution (5 µl) were added to 120 µl of 0.01–2.0 M glucose solution and incubated at room temperature for 1 h. The mixtures were added with 125 µl of 3 or 4 mM *p*NPG and 250 µl of McIlvaine buffer (pH 5.0) containing the same amount of glucose. The residual enzyme activity was measured at 80°C, pH 5.0 for 10 min. The *K*_i_ value was calculated by plotting the reciprocal of the reaction velocity and inhibitor concentration at each *p*NPG concentration.

### 2.10. HPLC analysis of the hydrolysis products of soybean four extract

To compare the hydrolysis efficiencies of NfBGL1 and Novozyme 188 towards isoflavone glycosides, 50 µl of 10% (w/v) soybean flour was incubated with 200 µl of each enzyme solution (0.05 U) in McIlvaine buffer (pH 5.0) in a thermostatically controlled incubator at 50°C. The reactions were terminated by ice water cooling. After centrifugation at 13,000 *g*, 4°C for 10 min, 20 µl of each supernatant was subject to HPLC analysis by using Waters HP1100 HPLC system (Milford, MA) equipped with a C18 column (5 µm, 4.6×250 mm). The solvent was composed of acetonitrile and 100 mM phosphate buffer (pH 5.0) at the ratio of 70∶30 (v/v) and run at a flow rate of 0.8 ml/min. The chromatograms were detected at 260 nm.

## Results and Discussion

### 3.1. Sequence analysis of *Nfbgl1*

The cDNA of *Nfbgl1* from *N. fischeri* P1 consists of 2220 base pairs and encodes a polypeptide of 739 amino acids with a putative signal peptide of 17 residues at the N-terminus. The molecular mass and *p*I value were estimated to be 78.8 kDa and 5.54, respectively. Two putative *N*-glycosylation sites (Asn207 and Asn381) were identified in deduced NfBGL1.

**Figure 1:**
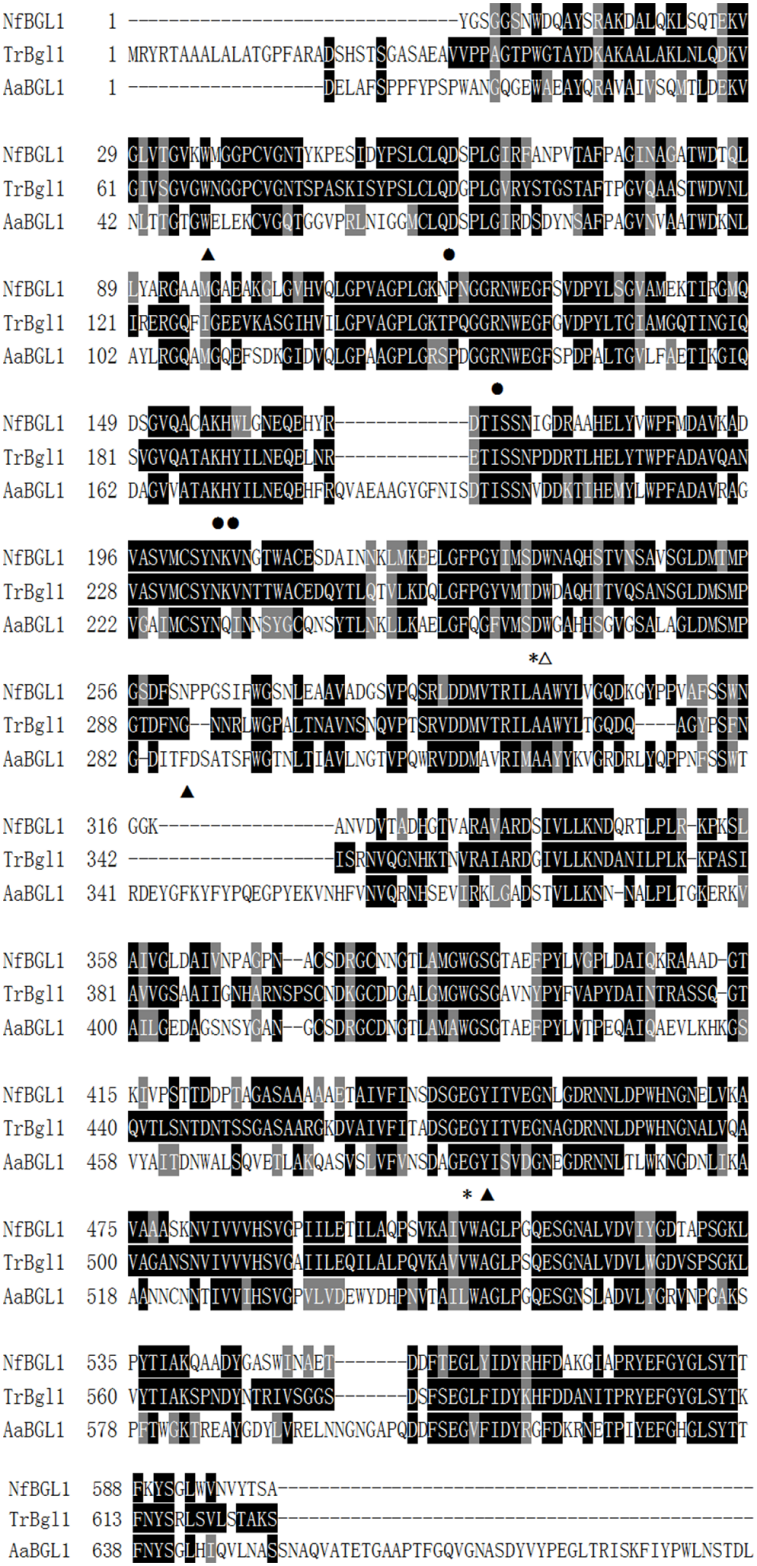
Multiple alignment of the deduced amino acid sequence of mature NfBGL1 (XP_001261562) with other fungal counterparts from *T. reesei* (TrBgl1, 1713235A), and *A. aculeatus* (AaBGL1, 4IIB). Identical and similar amino acids are indicated by solid black and gray boxes, respectively. The putative catalytic residues, D235 and E447, are indicated with asterisks. The residues probably related to subsite −1 (W236) and +1 (W36, N261, and Y449) are indicated with white and black triangles, respectively. The residues probably forming hydrogen bonds with substrate, D60, R124, K147 and H148, are indicated with black dots.

**Figure 2:**
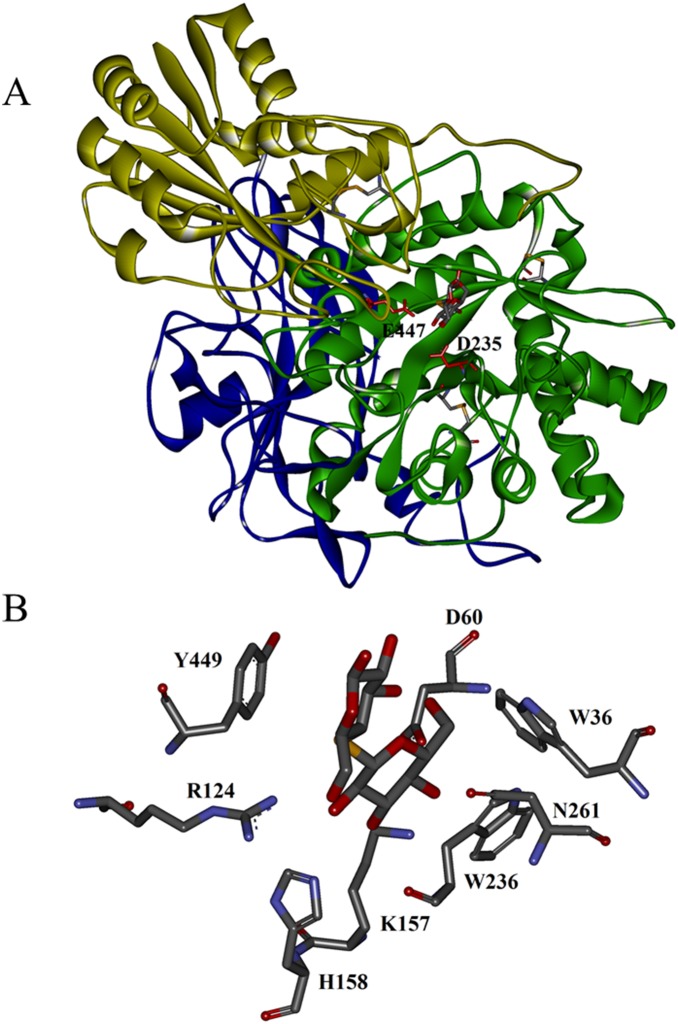
The homology-modeled NfBGL1 with AaBGL1 from *A. aculeatus* (4IIB, 44.1% identity) as the template. (A) Putative structure of NfBGL1. The catalytic residues D235 and E447 are indicated. (B) Putative interactions between the key residues of NfBGL1 and substrate cellobiose.

The deduced amino acid sequence of mature NfBGL1 showed the highest identity of 100% with the hypothetical β-glucosidase from *N. fischeri* NRRL 181 (XP_001261562), and of 61% identity with the *Trichoderma reesei* β-glucosidase (1713235A) with the experimentally evidenced activity [21]. Multiple sequence alignment and structure analysis with other β-glucosidases revealed that NfBGL1 is a typical β-glucosidase of GH3 (Fig. 1). Different from the two-domain barley β-glucosidase ExoI from *Hordeum vulgare* [22] and four-domain yeast β-glucosidase BglI from *Kluyveromyces marxianus* [23], the homology-based model of mature NfBGL1 with AaBGL1 as the template [16] was predicted to have three domains (Fig. 2A): the TIM barrel-like domain (Y1−S313), the α/β sandwich domain (T324−G526), and the C-terminal FnIII domain (Y585−W722). The putative catalytic residues, D235 in the barrel domain and E447 in the α/β sandwich domain, are located at the interface of the first two domains. Three disulfide bonds may form between C41–C57, C201–C212, and C374–C379 to stabilize the protein structure. Residues D60, R124, K157, and H158 are supposed to form hydrogen bonds with the sugar hydroxyls of substrate. Consistent with the structural features of AaBGL1, the subsite −1 of modeled NfBGL1 has a strict stereochemcial requirement, forming an aromatic stacking interaction with sugar ring by W236 (Fig. 2B) the equivalent of W281 of AaBGL1 [16]. The amino acid residues related to subsite +1 are less conserved, one (N261 of modeled NfBGL1) of the three residues (W36, N261 and Y449) is different. The diverse structures forming subsite +1 may account for the large substrate specificity ranges of GH3 β-glucosidases.

### 3.2. Overexpression, purification and high-cell-density production of NfBGL1

**Figure 3:**
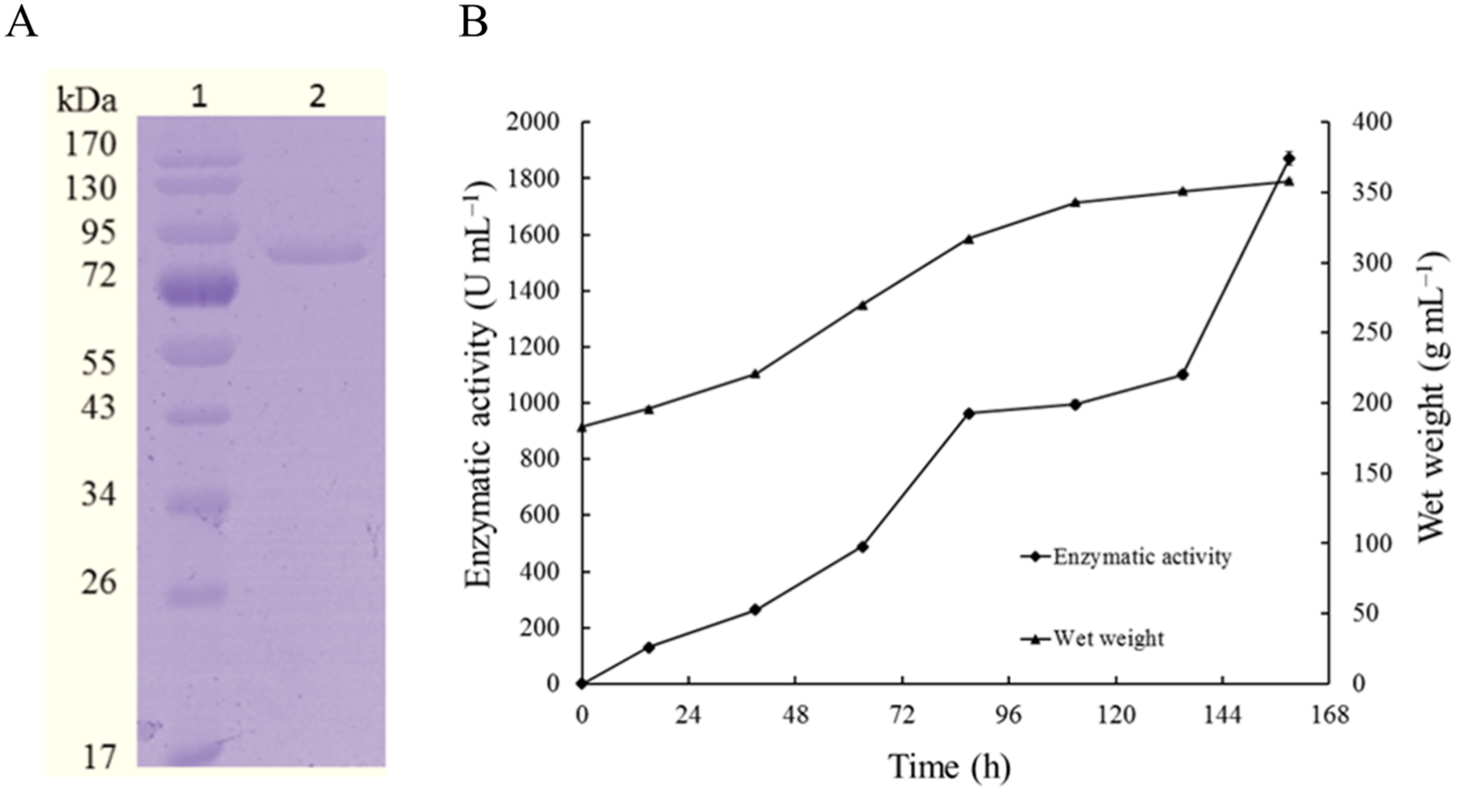
Purification and high-cell-density fermentation of recombinant NfBGL1. (A) SDS-PAGE analysis of purified recombinant NfBGL1. Lanes: 1, the molecular mass standards; 2, the purified recombinant of NfBGL1. (B) Time course of NfBGL1 production in a 3.7-l fermenter. Each value in the panel represents the means ± S.D. (*n* = 3).

The cDNA fragment of *Nfbgl1* without the signal peptide-coding sequence was amplified by PCR and cloned into the pPIC9 vector for heterologous expression. The β-glucosidase activity of recombinant NfBGL1 was determined to be 33.5±0.4 U/ml (pH 5.0, 50°C, 10 min) after 2-day growth in 1-l Erlenmayer flasks at 30°C. The culture supernatant was purified to electrophoretic homogeneity by one-step anion exchange chromatography. A single band of approximately 80 kDa migrated in SDS-PAGE (Fig. 3A), which is essentially identical to the calculated molecular weight of NfBGL1 (78.8 kDa). The result indicates that no *N*-glycosyaltion occurred in recombinant NfBGL1 during heterologous expression in *P. pastoris*. The specific activity of purified recombinant NfBGL1 against *p*NPG was 2189±1.7 U/mg, quite higher than that of all known fungal β-glucosidases (Table 1).

**Table 1:** Property comparison of NfBGL1 from *N. fischeri* P1 and its fungal counterparts. a

Microbial source	Optimum activity		Specific activity (U/mg)	*K*_m_ (mM)	*V*_max_ (µmol/min/mg)	*K*i for glucose (mM)	References
	pH	Temperature (°C)					
*N. fischeri* P1	5.0	80	2189	0.51	2172	13.4	This work
*Aspergillus terreus* NRRL265	5.0	60	-	2.5	-	13.6	[24]
*P. thermophila*	6.2	75	97.2	0.27	780	73	[25]
*Penicillium funiculosum* NCL1	4.0–5.0	60	1796	0.057	1920	1.5	[26]
*Trichoderma citrinoviride*	5.5	50	167	0.27	200	-	[27]
*Stachybotrys* sp.	6.0	50	-	1.85	211	-	[28]
*Fomitopsis palustris*	4.5	70	191	0.117	-	0.35	[29]
*Chaetomium thermophilum*	5.5	65	-	0.76	-	-	[30]
*Talaromyces emersonii*	4.0	71.5	483	0.13	512	0.254	[31]

a The substrate used for enzyme characterization is *p*NPG.

The transformant that showed the highest β-glucosidase activity in shaker flask cultures was subject to high-cell-density fermentation in a 3.7-l fermentor. The cell wet weight kept increasing, but no β-glucosidase activity was detected in the culture supernantant before the induction phase. Upon methanol induction, the β-glucosidase activity in the supernatant increased to a maximum of 1873±1.5 U/ml at 159 h, which was 56 fold of that of shake flask cultures (Fig. 3B). This significant activity increase might be ascribed to the higher dissolved oxygen levels and more efficient mass transfer in aerated system [32]. The high yield, easy purification and highest specific activity make NfBGL1 favorable for large-scale industrial production.

### 3.3. Enzymatic properties of purified recombinant NfBGL1

**Figure 4:**
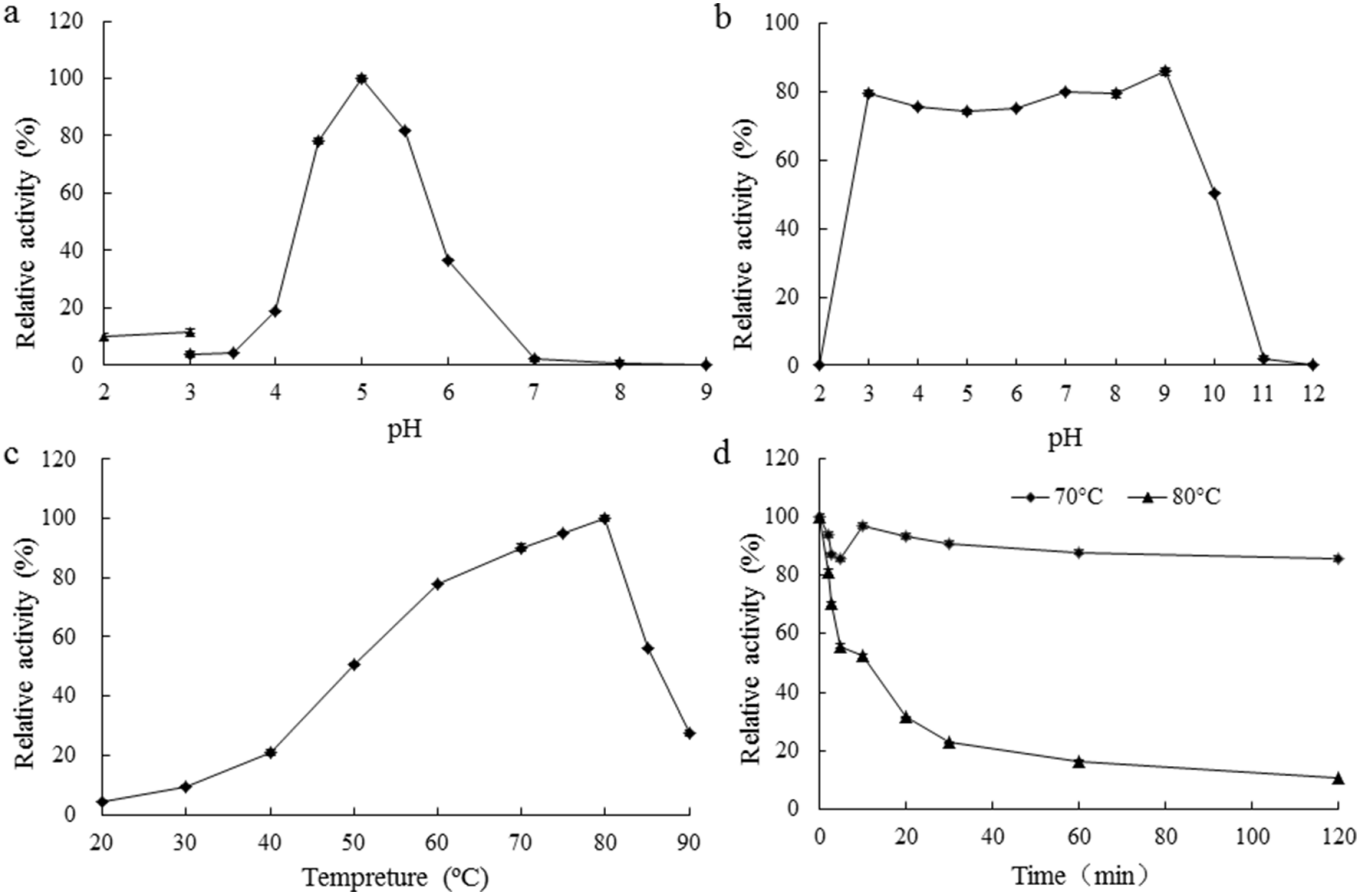
Characterization of purified recombinant NfBGL1. (A) Effect of pH on β-glucosidase activity. The enzyme assay was performed at 80°C for 10 min. (B) pH stability. The enzyme was pre-incubated without substrate at 37°C for 60 min, and then subjected to residual activity assay under standard conditions (pH 5.0, 80°C, 10 min). (C) Effect of temperature on β-glucosidase activity determined at pH 5.0 for 10 min. (D) Thermostability. The residual enzyme activities were measured under standard conditions after pre-incubation of the enzyme without substrate in McIlvaine buffer (pH 5.0) for various periods. Each value in the panel represents the means ± S.D. (*n* = 3).

Generally fungal β-glucosidases have an acidic pH optimum, ranging from 4.0 to 6.5, and are usually stable over a wide pH range (Table 1). NfBGL1 from *N. fischeri* P1 showed similar properties. It had a pH optimum of 5.0, and remained >80% of maximum activity at pH 4.5–5.5 (Fig. 4A). In comparison with other fungal counterparts, NfBGL1 was stable over a broader pH range, retaining >80% of the activity after incubation at pH 3.0 to 9.0, 37°C for 1 h (Fig. 4B). NfBGL1 represents a typical thermophilic/thermostable β-glucosidase. Of all characterized fungal β-glucosidases, that from *P. thermophila* [25] has the highest temperature optimum of 75°C, which is still 5°C lower than that of NfBGL1 (Fig. 4C). Moreover, NfBGL1 showed distinct adaptability and stability to high temperatures. It remained >50% of the activity at 50–85°C and 27% activity even at 90°C, and was thermostable at 70°C for 120 min (Fig. 4D). When increased the incubation temperature to 80°C, the enzyme retained 50% of the activity for 10 min and 11% activity for 120 min. NfBGL1 was resistant to most chemicals including Li^+^, Pb^2+^, Mn^2+^, Mg^2+^, Zn^2+^, Cr^3+^, Ni^+^, Cu^2+^, SDS, and EDTA, but was strongly inhibited by Ag^+^, Co^3+^, Fe^3+^, Ca^2+^ and β-mercaptoethanol (data not shown). Considering its acidic, thermophilic, wide pH/temperature range-stable and chemical-resistant advantages in biomass conversion, handling, post-processing and transportation [30], NfBGL1 has potentials for wide application in the biofuels, detergent, textile, and other industries.

### 3.4. Substrate specificity

NfBGL1 exhibited broad substrate specificity including β-glucosidase, cellobiase, glucanase, xylanase and isoflavone glycoside hydrolysis activities (Table 2). Besides *p*NPG, it was active towards the β-linked synthetic substrate *p*-nitrophenyl β-d-cellobioside and *p*-nitrophenyl β-d-xylopyranoside. Of all tested saccharides, sophorose (β-1,2-linked) was the most preferred substrate, followed by laminiarin (β-1,3, β-1,6-linked), salicin (β-linked), cellobiose (β-1,4-linked), lichenan (β-1,3, β-1,4-linked), and lactose (β-1,4-linked). Although most β-glucosidases have higher activities toward β-1,4-linked saccharides, NfBGL1 as well as the β-glucosidases from *Trichoderma koningii* [33] and a compost microbial metagenome [34] were found to be highly efficient in hydrolysis of β-1,2, 1,3, or 1,6-linkages. Such β-glucosidases may be involved in more biological activities than cellulose saccharification. Besides β-glucosidase and cellobiose activities, NfBGL1 displayed weak xylanase activities against beechwood xylan and birchwood xylan. No activity was detected against β-1,4-linked CMC-Na and Avicel. This broad substrate specificity suggests that NfBGL1 may have loose stereochemcial specificity at subsite +1 as indicated by the structure analysis described above. Thus NfBGL1 can be used in various biotechnological fields for diverse saccharide degradation.

**Table 2:** Substrate specificities of NfBGL1.

Substrate	Specific activity(U/mg)^a^	Substrate	Specific activity (U/mg)
*p*NPG	2189±1.7	Cellobiose	203.8±0.9
*p*-Nitrophenyl β-d-cellobioside	601.0±0.9	Lichenin	93.3±1.2
*p*-Nitrophenyl β-d-xylopyranoside	6.6±0.3	Daidzin	90.1±0.7
*p*-Nitrophenyl β-d-galactoside	–	Glycitin	75.2±0.6
*p*-Nitrophenyl α-l-arabinofuranoside	–	Genistin	45.6±0.3
*p*-Nitrophenyl α-l-arabinopyranoside	–	Beechwood xylan	6.4±0.5
Sophorose	896.1±0.9	Birchwood xylan	6.2±0.7
Laminiarin	675.4±1.1	Lactose	3.5±0.3
Gentiobiose	602.4±0.8	Xyloglucan	–
Amygdalin	510.8±0.7	Avicel	–
Barley β-glucan	269.4±1.3	CMC-Na	–
Salicin	246.6±0.6		

a Values are means ± S.D. (*n* = 3); –, no activity detected.

### 3.5. Kinetic analysis and tolerance to glucose inhibition

With *p*NPG as the substrate, the *K*_m_, *V*_max_ and *k*_cat_ values for NfBGL1 was 0.51 mM, 2172 µmol/min/mg, and 2853/s, respectively. The *K*_m_ value of NfBGL1 fell within the range of fungal β-glucosidase *K*_m_ values, but its *V*_max_ value was quite high (Table 1). Catalytic efficiency (*k*_cat_ */K*_m_) is the true kinetic parameter to evaluate enzyme catalytic performance. The *k*_cat_ */K*_m_ value of NfBGL1 was 5594/mM/s, higher than that of β-glucosidases from *P. thermophila* (42.8/mM/s) [25], *Stachybotrys* sp. (494.6/mM/s) [28], and *C. thermophilum* (193.4/mM/s) [30].

Glucose accumulation during enzymatic hydrolysis can significantly lower the cellulose hydrolysis rate by blocking the active site or preventing the release of hydrolysis products [35]. Thus high tolerance towards glucose accumulation is a requirement of fungal β-glucosidases. To our best knowledge, the GH3 β-glucosidases from *Candida peltata* [36] and *Aspergillus oryzae* [37] exhibited the strongest glucose tolerance with the *K*i values of 1.4 and 1.36 M, respectively. Compared with these two fungal counterparts, NfBGL1 showed less resistance to glucose inhibition with a *K*i of 13.4 mM, but the glucose tolerance was much higher than the β-glucosidases from *T. reesei*, *F. palustris*, and *P. funiculosum* NCL1 (Table 1).

### 3.6. Hydrolysis of soybean isoflavone glycosides by NfBGL1

β-Glucosidases with hydrolysis ability to degrade soybean isoflavone glycosides have application potential in food and feed industry. Based on the HPLC analysis, the soybean flour extract contained both isoflavone glucosides (270 mg/g; daidzin, genistin and glycitin) and isoflavone aglycones (343 mg/g; daidzein, genistein and glycitein) (Table 3). After 5-min incubation at 50°C, the contents of daidzin, genistin and glycitin were remarkably decreased, which suggested that most soybean isoflavone glucosides (>90%) were converted to free isoflavones by NfBGL1. The conversion efficiency of daidzin and glycitin reached up to 100%, and 90% for the isoflavone glucosides tested. Under the same conditions, the commercial β-glucosidase Cellobiase (Novozyme 188) was a little less efficient in isoflavone glycoside hydrolysis, converting 82% of glycosides to aglycones. In addition, more free isoflavonones were detected in the soybean flour extract after treatment with Cellobiase than with NfBGL1 (1120 mg/g v.s. 920 mg/g). It might be ascribed to the presence of other unidentified soybean isoflavone glycosides like malonyl or acetyl.

**Table 3:** Conversion of soybean isoflavone glycosides into free isoflavones by NfBGL1 and Novozyme 188. a

Enzyme	Isoflavone glycosides (mg/g)			Free isoflavonones (mg/g)		
	Daidzin	Genistin	Glycitin	Daidzein	Genistein	Glycitein
Control	146.8±0.4	68.7±0.3	54.0±0.2	100.8±0.7	220.7±1.1	21.7±0.4
NfBGL1	0	25.9±0.4	0	222.4±0.9	598.2±0.6	73.4±1.0
Novozyme 188	0	32.7±0.6	16.9±0.1	253.6±0.8	746.5±1.0	70.3±1.1

a The reaction system without enzyme addition was treated as the control; the data are shown as mean ± S.D. (*n* = 3).

## Conclusion

In the present study, a highly-active, thermophilic, soybean isoflavone glycoside-degrading β-glucosidase of GH3 was identified in *N. fischeri* P1. The enzyme had an optimum temperature and specific activity higher than that of all known fungal counterparts, was stable over a broader pH and temperature ranges, and had strong resistance to most chemicals tested. Its substrate specificity was remarkably broad, including glucosidase, cellobiase, xylanase and glucanase activities. Its high efficiency to convert isoflavone glycosides of different soybean products into aglycons was much attractive. These characteristics make it a preeminent enzyme candidate in the food, feed, pharmacy, and biofuel industries.
